# Temporal Trends and Outcomes of Percutaneous and Surgical Aortic Valve Replacement in Patients With Atrial Fibrillation

**DOI:** 10.3389/fcvm.2020.603834

**Published:** 2020-12-07

**Authors:** Jing Wu, Chenguang Li, Yang Zheng, Qian Tong, Quan Liu, Xiaoqiang Cong, Zhiyang Lou, Mingyou Zhang

**Affiliations:** ^1^Department of Cardiovascular Medicine, The First Hospital of Jilin University, Changchun, China; ^2^Department of Cardiovascular Medicine, Zhongshan Hospital, Shanghai, China

**Keywords:** AF, in-hospital mortality, SAVR, TAVR, NIS

## Abstract

**Objectives:** The aim of this study was to evaluate the temporal trends of transcatheter aortic valve replacement (TAVR) in severe aortic stenosis (AS) patients with atrial fibrillation (AF) and to compare the in-hospital outcomes between TAVR and surgical aortic valve replacement (SAVR) in patients with AF.

**Background:** Data comparing TAVR to SAVR in severe AS patients with AF are lacking.

**Methods:** National inpatient sample database in the United States from 2012 to 2016 were queried to identify hospitalizations for severe aortic stenosis patients with AF who underwent isolated aortic valve replacement. A propensity score-matched analysis was used to compare in-hospital outcomes for TAVR vs. SAVR for AS patients with AF.

**Results:** The analysis included 278,455 hospitalizations, of which 124,910 (44.9%) were comorbid with AF. Before matching, TAVR had higher in-hospital mortality than SAVR (3.1 vs. 2.2%, *p* < 0.001); however, there was a declining trend during the study period (Ptrend < 0.001). After matching, TAVR and SAVR had similar in-hospital mortality (2.9 vs. 2.9%, *p* < 0.001) and stroke. TAVR was associated with lower rates of acute kidney injury, new dialysis, cardiac complications, acquired pneumonia, sepsis, mechanical ventilation, tracheostomy, non-routine discharge, and shorter length of stay; however, TAVR was associated with more pacemaker implantation and higher cost. Of the patients receiving TAVR, the presence of AF was associated with an increased rate of complications and increased medical resource usage compared to those without AF.

**Conclusions:** In-hospital mortality and stroke for TAVR and SAVR in AF, AS are similar; however, the in-hospital mortality in TAVR AF is declining and associated with more favorable in-hospital outcomes.

## Prospectives

WHAT IS KNOWN? Data comparing TAVR to SAVR in severe AS patients with AF are lacking.

WHAT IS NEW? In this national analysis, TAVR and SAVR have similar in-hospital mortality for AS patients with AF, however, there was a declining trend in TAVR during the study period. TAVR was associated with a lower rate of blood transfusion, cardiac complications, acute kidney injury, new dialysis, acquired pneumonia, sepsis, mechanical ventilation, tracheostomy, and gastrostomy, as well as more routine discharge and shorter length of hospitalization stay; however, TAVR increased permanent pacemaker implantation and medical cost. The presence of AF was associated with more complications and medical resource usage.

WHAT IS NEXT? AF should be an important factor to consider when deciding on optimal treatment and long-term management.

## Introduction

Patients with aortic stenosis (AS) who are candidates for aortic valve replacement (AVR) are fundamentally an aging population, and the prevalence of atrial fibrillation (AF) among these patients is markedly high (1, 2). As the aging population is expanding worldwide, the number of patients with AF who undergo AVR is increasing. Compared to surgical aortic valve replacement (SAVR), transcatheter aortic valve replacement (TAVR) is less invasive and significantly reduces hospital stays. Data from randomized clinical trials as well as real-world observational data have shown that TAVR is an effective treatment for AS. Following the evidence from PARTNER, PARTNER 2, PARTNER 3, CoreValve High Risk Study, SURTAVI, and Evolut Low Risk, TAVR is an appealing alternative for SAVR across all surgical risk populations (3–8). AF is a well-established predictor of adverse outcomes in patients treated with SAVR (2). In the TAVR era, AF was also found to be related to an increased risk for adverse outcomes, including increased mortality and stroke (9, 10). There are limited studies comparing the outcome of TAVR vs. SAVR specifically in AF patients. The current study represents the largest real-world cohort to report in-hospital outcomes with TAVR vs. SAVR in patients with AF. This study evaluates the in-hospital outcomes of AF patients comparing TAVR with SAVR, as well as the impact of AF on TAVR patients compared to patients without AF in a large cohort of hospitalizations from the Nationwide Inpatient Sample (NIS).

## Methods

### Data Source

This study cohort was derived from the NIS database, which is the largest publicly available inpatient care database in the United States. NIS database containing more than 100 clinical data elements from over seven million hospital stays annually, representing 20% of hospital admissions in the United States (11–16). In the current analysis, data before September 2015 were coded with the International Classification of Diseases-9th Revision (ICD-9), and after September 2015, data were coded with the International Classification of Diseases-10th Revision (ICD-10). The datasets contain unidentified, publicly available data; therefore, this study was exempt from the Institutional Review Board evaluation.

### Study Population and Outcome

We queried the NIS database (2012–2016) to identify patients with ICD-9 or ICD-10 procedural codes for TAVR or SAVR. We excluded hospitalization with concomitant mitral, tricuspid, or pulmonary valve procedures, excluded patients younger than 50 years old, excluded concomitant coronary artery bypass grafting (CABG) to identify isolated aortic valve replacement, and excluded hospitalizations with missing data for mortality outcomes or propensity matching variables. To identify patients with AF, ICD-9 diagnostic codes (427.31) and ICD-10 diagnostic codes (I48, I48.0, I48.1, I48.11, I48.19, I48.2, I48.20, I48.21, and I48.91) were used.

The main aim of this study was to compare the temporal trends and outcomes of TAVR and SAVR in hospitalizations in patients with AF. A secondary analysis to compare the outcomes of TAVR in patients with AF vs. patients without AF was also conducted. The primary outcome was in-hospital mortality. Secondary outcomes included acute stroke, blood transfusion, acute kidney injury, new dialysis, permanent pacemaker implantation, cardiac complications, tamponade, acquired pneumonia, urinary tract infection, sepsis, over 96 h of mechanical ventilation, tracheostomy, gastrostomy, cardiac arrest, cardiac shock, discharge to a nursing facility, length of hospital stay, and medical cost. A detailed list of the ICD-9, ICD10, and clinical classification codes used as reported by the Healthcare Cost and Utilization Project are reported in Supplementary Table 1.

### Statistical Analyses

We used propensity scores to match patients with AF who underwent TAVR to those who underwent SAVR at a 1:1 ratio. We used the MatchIt R package nearest neighbor technique with a caliper width of 0.1 to perform propensity score matching (17). The following 23 variables were used for propensity score calculation: age, race, sex, hypertension, diabetes mellitus, diabetes with chronic complications, chronic kidney disease (CKD), chronic lung disease, chronic anemia, chronic arthritis, coagulopathy, hypothyroidism, chronic liver disease, obesity, weight loss, peripheral artery disease, chronic pulmonary circulatory disorder, fluid or electrolyte disturbance, median household income for the patient's ZIP Code, elective admission, insurance status, hospital bed size, and hospital teaching status. The NIS data were merged with cost-to-charge ratios available from the Healthcare Cost and Utilization Project to estimate the cost of hospitalization. The cost of each hospitalization was estimated by multiplying the total hospital charge by the cost-to-charge ratio. We conducted a multivariable analysis to identify clinical and hospital characteristics independently associated with TAVR among patients with AF. We also conducted Pre-specified subgroup analyses for in-hospital mortality within the TAVR and SAVR groups. A cohort after propensity matching was used for the subgroup analysis to maintain the balance between TAVR and SAVR.

We conducted a secondary analysis to compare TAVR patients with AF vs. patients without AF. Similar to the main propensity-matched model, a propensity score was used to match patients with AF who underwent TAVR to those without AF who underwent TAVR. The same 23 variables were used for propensity score matching, and the same caliper width of 0.1 was used to perform the matching. All analyses were conducted using the weighting samples for national estimates in conjunction with the Healthcare Cost and Utilization Project regulations for using the NIS database (18). Categorical variables were assessed using the chi-square test, and continuous variables using the Student's *t*-test. We calculated temporal trending using the Cochran-Armitage trend test. Categorical variables, such as proportions, and continuous variables were reported as the mean ± *SD* or median (interquartile range [IQR]) whenever appropriate. We used the Breslow-Day test to measure the homogeneity of the odds ratio (OR) for the subgroup analyses. Effect sizes were showed with OR and 95% confidence intervals (CI). A *p* < 0.05 was considered significant, calculated using the SAS 9.4 (SAS Institute Inc., Cary, North Carolina) and R software version 3.5.2 (R Foundation for Statistical Computing, Vienna).

## Results

### Population Characteristics

**Figure 1:**
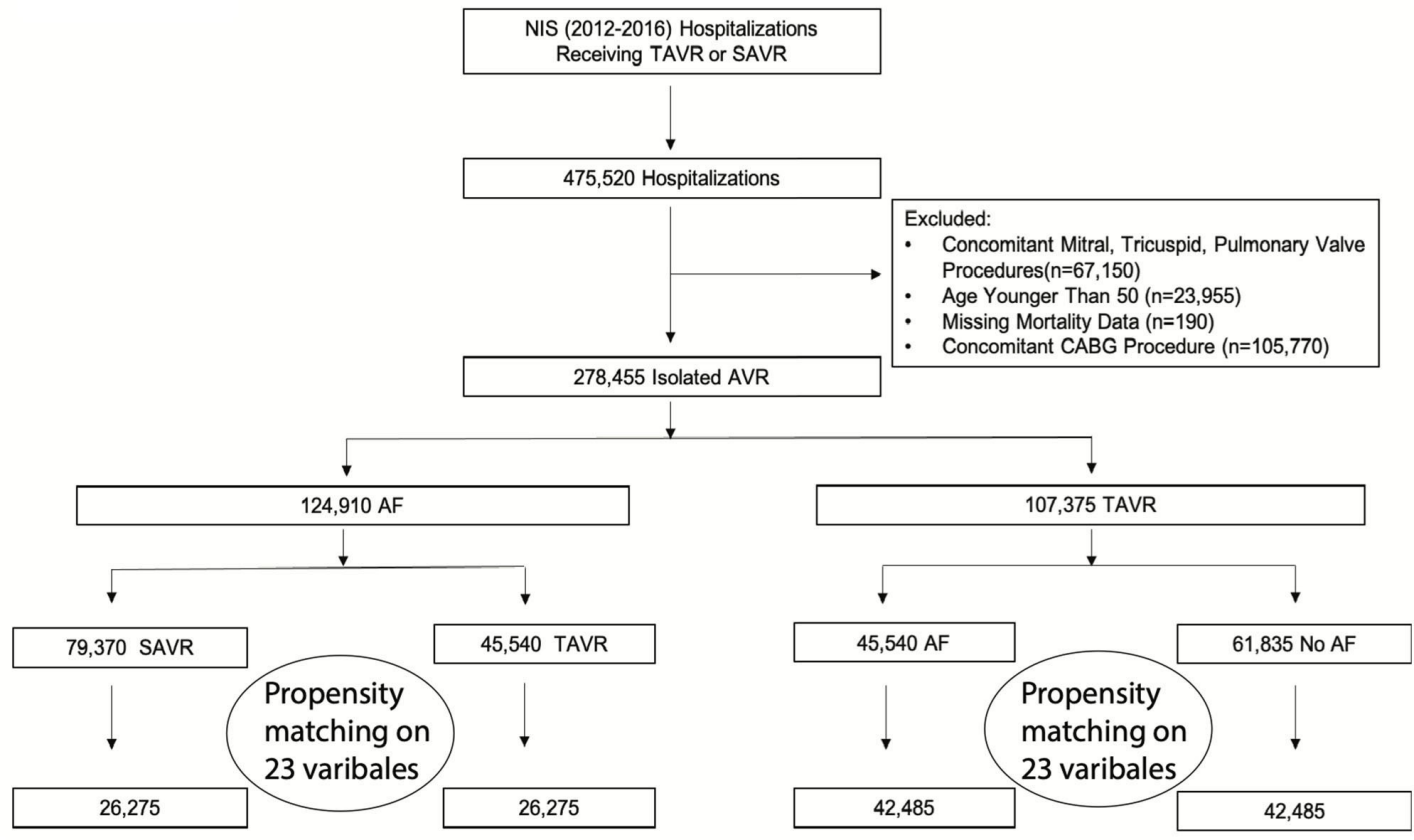
Study flow chart. AF, atrial fibrillation; AS, aortic stenosis; AVR, aortic valve replacement; CABG, coronary artery bypass grafting; NIS, national inpatient sample; SAVR, surgical aortic replacement; TAVR, transcatheter aortic valve replacement.

The study flow chart is outlined in Figure 1. From 2012 to 2016, the initial analysis yielded 475,520 hospitalizations that underwent SAVR or TAVR. After excluding patients with other valvular surgeries, concomitant CABG, age younger than 50, or missing variables, we included 278,455 hospitalizations. Among those, 124,910 (44.9%) had AF, which represented the final cohort. A total of 45,540 (36.5%) patients underwent TAVR, and 79,370 (63.5%) underwent SAVR. After propensity score matching, there were 52,550 hospitalizations: among those, 26,275 in the TAVR group and 26,275 in the SAVR group. Overall, the trend for performance of aortic valve replacement (AVR) procedures during the study period was rising. In patients with AF, the rising trend of AVR was driven by rising TAVR performance. The performance of SAVR was similar during the study period; however, the rising trend of TAVR in AF patients was slower than that without AF (Supplementary Figure 1).

The study cohort baseline characteristics are presented in Table 1. Before propensity matching, patients in the TAVR group were significantly older (81.9 ± 7.2 years of age vs. 72.1 ± 9.2 years of age; *p* < 0.001) and less likely to be men (53.9% vs. 62.0%; *p* < 0.001) compared with patients underwent SAVR. The patients who underwent TAVR associated more comorbidities, including diabetes, diabetes with chronic complications, chronic lung disease, congestive heart failure, chronic renal disease, anemia, arthritis, coagulopathy, hypothyroidism, chronic liver disease, obesity, peripheral vascular disease, and pulmonary circulation disorder. Additionally, patient in urban, large, and teaching hospitals are more likely underwent TAVR. After propensity score matching, the baseline characteristics of the study cohort were similar indicated by the standardized mean differences for all matching variables were <10% between TAVR and SAVR groups.

**Table 1:** Baseline Characteristics for TAVR and SAVR in the Unmatched and Matched Cohorts.

	**Unmatched Cohort**		***P*-value**	**Matched Cohort**	
	**TAVR in AF** ** (*n* = 45,540)**	**SAVR in AF** ** (*n* =79,370)**		**TAVR in AF** ** (*n* = 26,275)**	**SAVR in AF** ** (*n* = 26,275)**
Age, yrs	81.9 ± 7.2	72.1± 9.2	<0.001	79.3 ± 7.6	78.9 ± 6.6
Female	46.1	38	<0.001	44.2	44.4
Race			<0.001		
White	90	87.3		88.9	88.0
Black	2.7	3.5		3.2	3.2
Hispanic	3.1	5		3.3	4.7
Hypertension	75.5	75.3	0.78	75.9	75.7
Diabetes	25.2	22.9	<0.001	25.6	25.2
Diabetes with chronic complications	8.3	6.7	<0.001	8.2	8.5
Chronic lung disease	32.2	21.7	<0.001	28.7	28.1
Congestive heart failure	5.6	1.5	<0.001	4.6	4.4
Chronic renal disease	36.5	16.5	<0.001	29.0	27.0
Anemia	25.4	18.5	<0.001	20.2	19.6
Arthritis	4.6	3.2	<0.001	4.3	4.1
Coagulopathy	23.8	36.3	<0.001	24.8	25.5
Hypothyroidism	21.4	14.1	<0.001	17.1	16.8
Liver disease	2.1	1.6	0.04	2.1	2.1
Obesity	14	22	<0.001	16.5	16.5
Weight loss	5.4	5.2	0.58	5.0	4.9
Peripheral vascular disease	30.2	16.5	<0.001	22.0	21.7
Pulmonary circulation disorder	3.1	0.7	<0.001	1.0	0.9
Teaching hospital	89.9	74.5	<0.001	85.7	85.3
Rural location	0.9	2.4	<0.001	1.4	0.6
Large hospital bed size	77.3	71.0	<0.001	73.5	73.8
**Primary payer**					
Medicare/Medicaid	92.6	79.1	<0.001	91.8	91.5
Private insurance	5.6	18.0	<0.001	6.5	6.9
Non-elective admission	24	27.4	<0.001	76.3	76.7
0–25th percentile income	19.5	20.6	0.07	20.9	20.9

*Values are mean ± SD or percentages*.

*AF, atrial fibrillation; SAVR, surgical aortic valve replacement; TAVR, transcatheter aortic valve replacement*.

### Predictors of TAVR in Patients With AF

**Figure 2:**
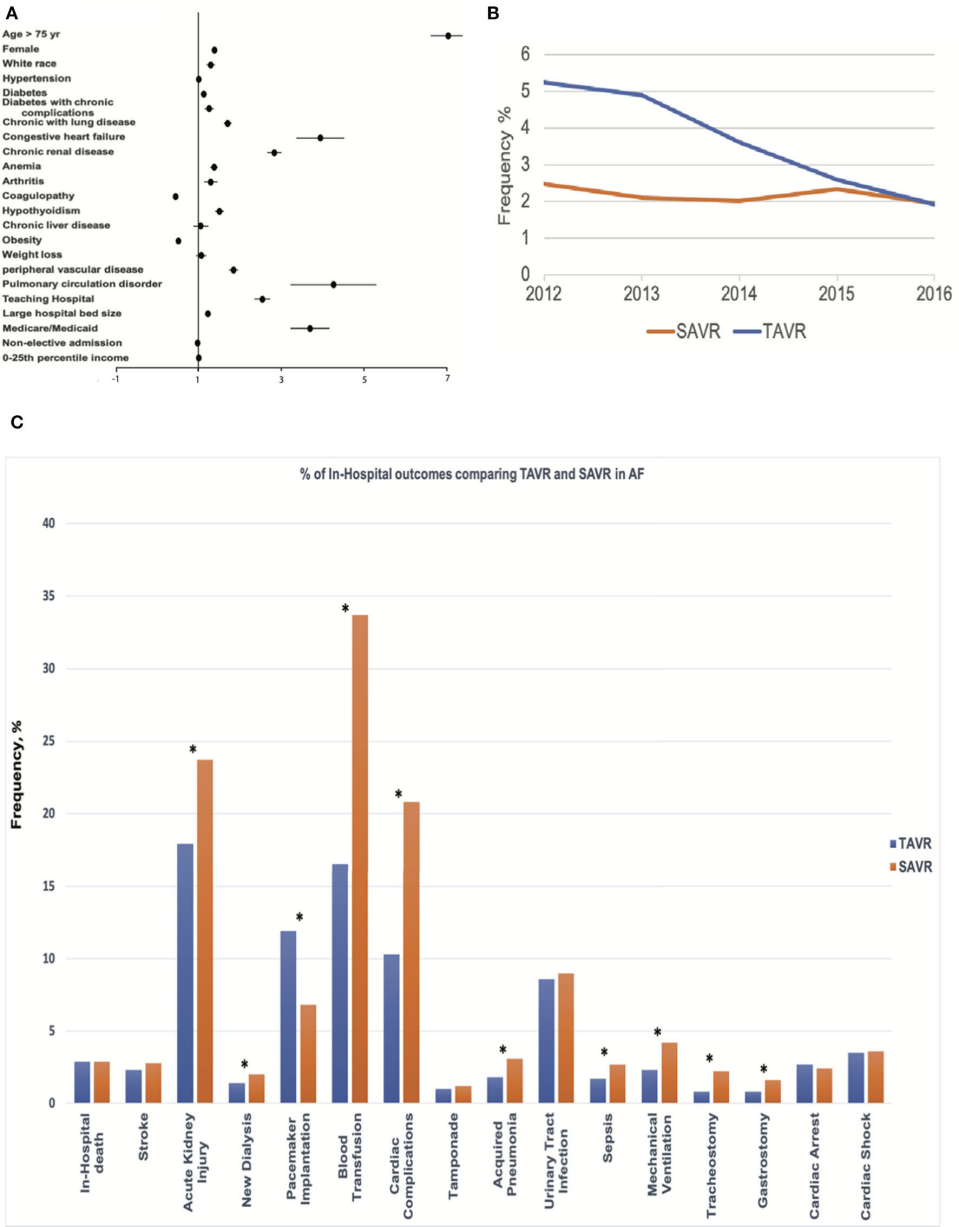
Central Illustration. Mortality trend, Predictor, and Outcomes of TAVR for Severe Aortic Stenosis with AF. **(A)** Clinical and hospital predictors of undergoing transcatheter aortic valve replacement (TAVR) procedures. **(B)** The trend in mortality of TAVR and surgical aortic replacement (SAVR) procedures for severe aortic stenosis (AS) patients with AF. **(C)** Comparative outcomes between TAVR and SAVR in AF among the propensity-matched cohort. *indicates *p* < 0.05.

In the AF cohort, certain clinical predictors were independently associated with undergoing TAVR (Figure 2A), including older age; the odds ratio (OR) for undergoing TAVR was 7.05 with a 95% CI 6.62–7.51 when comparing AF patients older than 75 to younger or equal to 75. TAVR was more likely in females (OR: 1.39; 95% CI: 1.32–1.47), whites (OR: 1.30; 95% CI: 1.20–1.43), and several comorbidities, including diabetes (OR: 1.13; 95% CI: 1.06–1.20), comorbid with diabetes with chronic complication (OR: 1.26; 95% CI: 1.15–1.39), chronic lung disease (OR: 1.71; 95% CI: 1.62–1.81), congestive heart failure (OR: 3.95; 95% CI: 3.37–4.62), chronic renal disease (OR: 2.84; 95% CI: 2.67–3.02), anemia (OR: 1.38; 95% CI: 1.29–1.47), arthritis (OR: 1.30; 95% CI: 1.14–1.49), hypothyroidism (OR: 1.51; 95% CI: 1.41–1.62), peripheral vascular disease (OR: 1.85; 95% CI: 1.74–1.97), and pulmonary circulation disorder (OR: 4.27; 95% CI: 3.23–5.65). In contrast, comorbidities with obesity (OR: 0.52; 95% CI: 0.49–0.56) and coagulopathy (OR: 0.45; 95% CI: 0.43–0.48) are less likely to undergo TAVR. Teaching hospital (OR: 2.55; 95% CI: 2.36–2.76), large hospital bed size (OR: 1.23; 95% CI: 1.16–1.31), and Medicare or Medicaid coverage (OR: 3.7; 95% CI: 3.45–4.17) are also factors associated with undergoing TAVR.

### Study Outcomes

TAVR was associated with higher in-hospital mortality compared with SAVR (3.1 vs. 2.2%; *p* < 0.001) before propensity matching. In-hospital mortality in TAVR was declining during the study period (decreasing from 5.24% in 2012 to 1.92% in 2016, Ptrend < 0.001), while mortality in SAVR remained unchanged (Ptrend = 0.31) (Figure 2B). TAVR was associated with more permanent pacemaker implantation (11.9 vs. 6.4%; *p* < 0.001), urinary tract infection (8.9 vs. 6.7%; *p* < 0.001), cardiac arrest (2.7 vs. 2.1%; *p* < 0.001), and higher hospitalization cost (57,178 ± 29,547 vs. 50,642 ± 31,853; *p* < 0.001); however, TAVR was associated with less acute kidney injury (18.1 vs. 19.5%; *p* < 0.001), blood transfusion (16.3 vs. 30.1%; *p* < 0.001), cardiac complication (9.9 vs. 22.1%; *p* < 0.001), acquired pneumonia (1.8 vs. 2.8%; *p* < 0.001), sepsis (1.6 vs. 2.7%; *p* < 0.001), mechanical ventilation (2.1 vs. 3.6%; *p* < 0.001), tracheostomy (0.7 vs. 1.5%; *p* < 0.001), gastrostomy (0.8 vs. 1.1%; *p* < 0.001), shorter length of stay [5 (IQR: 3–9) vs. 8 (IQR: 6–11)], and more routine discharge (38.4 vs. 30.4%; *p* < 0.001) (Table 2).

**Table 2:** Outcomes for TAVR and SAVR in the Unmatched and Matched Cohorts.

	**Unmatched Cohort**		***p***	**Matched Cohort**		***p***
	**TAVR in AF**	**SAVR in AF**		**TAVR in AF**	**SAVR in AF**	
In-Hospital death	3.1	2.2	<0.001	2.9	2.9	0.91
Stroke	2.6	2.5	0.83	2.3	2.8	0.12
Acute kidney injury	18.1	19.5	<0.001	17.9	23.7	<0.001
New dialysis	1.5	1.7	0.25	1.4	2	0.04
Pacemaker implantation	11.9	6.4	<0.001	11.9	6.8	<0.001
Blood transfusion	16.3	30.1	<0.001	16.5	33.7	<0.001
Cardiac complications	9.9	22.1	<0.001	10.3	20.8	<0.001
Tamponade	1.0	1.3	0.07	1	1.2	0.36
Acquired pneumonia	1.8	2.8	<0.001	1.8	3.1	<0.001
Urinary tract infection	8.9	6.7	<0.001	8.6	9	0.54
Sepsis	1.6	2.7	<0.001	1.7	2.7	<0.001
Mechanical ventilation	2.1	3.6	<0.001	2.3	4.2	<0.001
Tracheostomy	0.7	1.5	<0.001	0.8	2.2	<0.001
Gastrostomy	0.8	1.1	0.02	0.8	1.6	<0.001
Cardiac arrest	2.7	2.1	0.002	2.7	2.4	0.46
Cardiac Shock	3.3	3.8	0.05	3.5	3.6	0.83
Discharge status			<0.001			<0.001
Routine	38.4	30.4		41	20.9	
Home Health Care	29.8	36.2		30	32.2	
Other Care Facility	28	30.3		25.1	42.9	
Length of stay, days	5 (3, 9)	8 (6, 11)	<0.001	5 (3, 9)	8 (6, 12)	<0.001
Mean cost, $	57,178 ± 29,547	50,642 ± 31,853	<0.001	57,230 ± 32,279	51,271 ± 31,811	<0.001

Values are mean ± SD, median (interquartile range), or percentages.

SAVR, surgical aortic valve replacement; TAVR, transcatheter aortic valve replacement.

**Figure 3:**
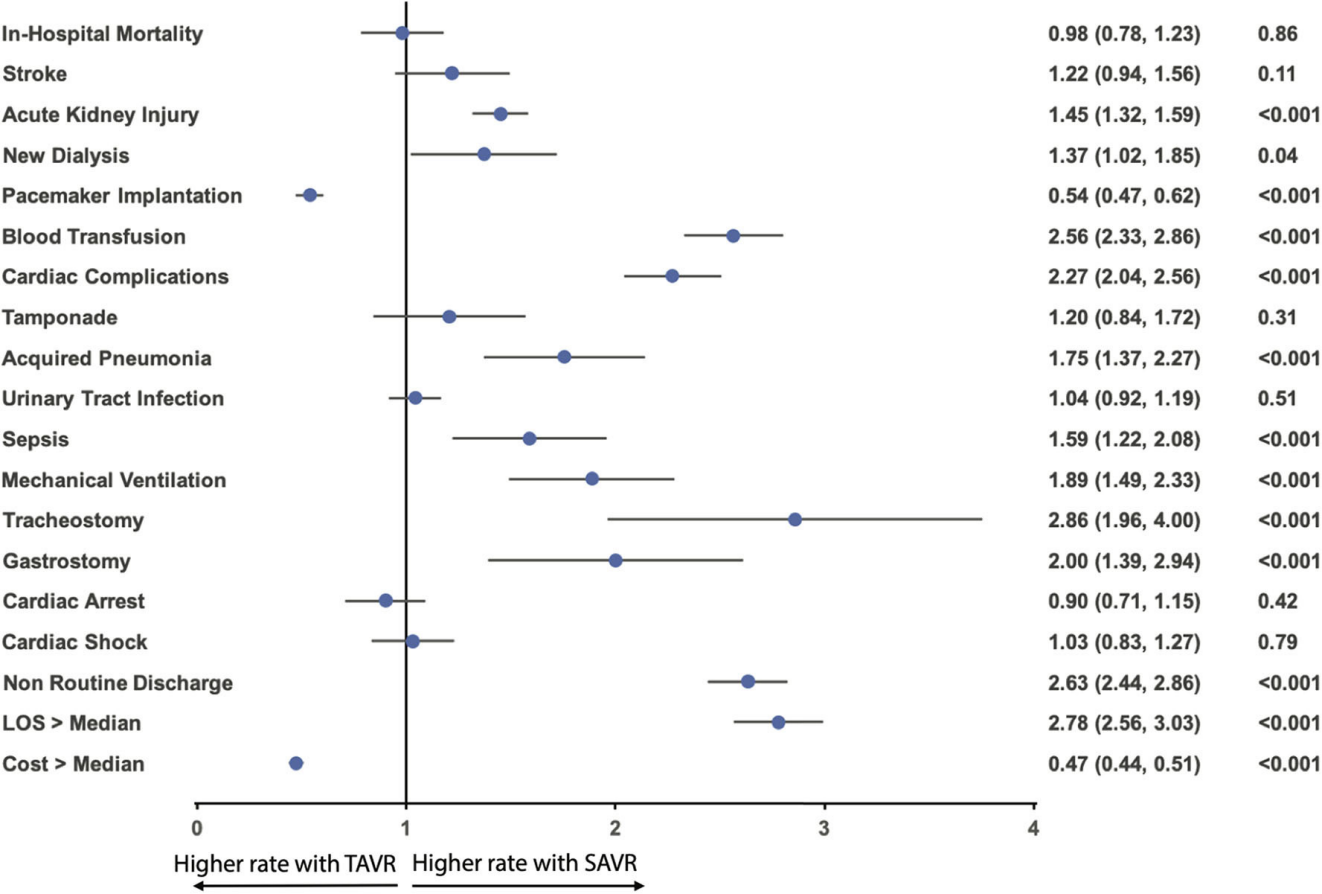
Odds ratio forest plot for in-hospital outcomes comparing TAVR vs. SAVR in AF AS among the matched cohort. LOS, length of hospital stay; other abbreviations as in Figure 1.

The in-hospital mortality between TAVR and SAVR was similar after propensity matching (2.9 vs. 2.9%; OR:1.02; 95% CI: 0.81–1.28; *p* = 0.91) (Figure 2C), as well as acute stroke (2.3 vs. 2.8%; OR:0.82; 95% CI: 0.64–1.06; *p* = 0.12), tamponade (1.0 vs. 1.2%; OR:0.83; 95% CI: 0.58–1.19; *p* = 0.36), urinary tract infection (8.6 vs. 9.0%; OR:0.96; 95% CI: 0.84–1.09; *p* = 0.54), cardiac arrest (2.7 vs. 2.4%; OR:1.11; 95% CI: 0.87–1.41; *p* = 0.46), and cardiac shock (3.5 vs. 3.6%; OR:0.97; 95% CI: 0.79–1.20; *p* = 0.83). However, TAVR was associated with a lower rate of acute kidney injury (17.9 vs. 23.7%; OR:0.69; 95% CI: 0.63–0.76; *p* < 0.001), new dialysis (1.4 vs. 2.0%; OR:0.73; 95% CI: 0.54–0.98; *p* = 0.04), blood transfusion (16.5 vs. 33.7%; OR:0.39; 95% CI: 0.35–0.43; *p* < 0.001), cardiac complication (10.3 vs. 20.8%; OR:0.44; 95% CI: 0.39–0.49; *p* < 0.001), acquired pneumonia (1.8 vs. 3.1%; OR:0.57; 95% CI: 0.44–0.73; *p* = 0.42), sepsis (1.7 vs. 2.7%; OR:0.63; 95% CI: 0.48–0.82; *p* < 0.001), mechanical ventilation (2.3 vs. 4.2%; OR:0.53; 95% CI: 0.43–0.67; *p* < 0.001), tracheostomy (0.8 vs. 2.2%; OR:0.35; 95% CI: 0.25–0.51; *p* < 0.001), gastrostomy (0.8 vs. 1.6%; OR:0.5; 95% CI: 0.34–0.72; *p* < 0.001), shorter hospital stay [5 (IQR: 3–9) in TAVR vs. 8 (IQR: 6–12) in SAVR], and more routine discharge (40.0 vs. 20.9%; OR:2.63; 95% CI: 2.44–2.86; *p* < 0.001). However, TAVR was still associated with higher permanent pacemaker implantation (11.9 vs. 6.8%; OR: 1.86; 95% CI: 1.62–2.13; *p* < 0.001) and hospitalization cost (57,2s30 ± 32,279 vs. 51,271 ± 31,811; *p* < 0.001) (Table 2 and Figure 3).

**Figure 4:**
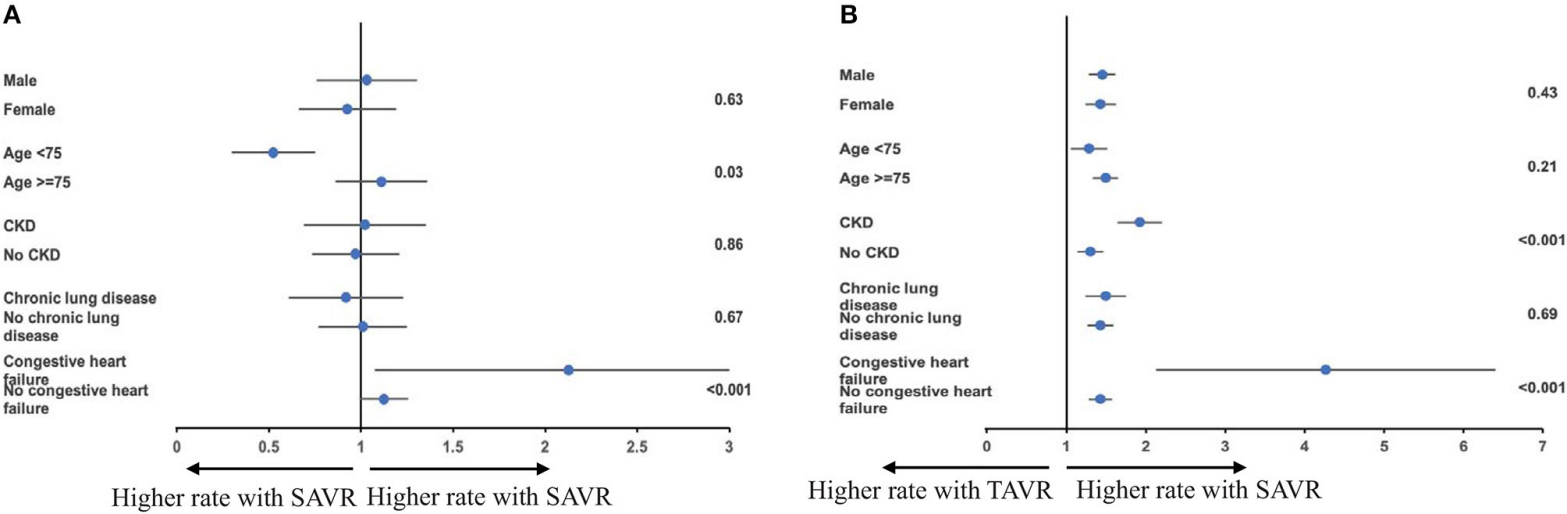
**(A,B)** Subgroup analysis for mortality and acute kidney injury among the matched cohort according to sex, age, chronic kidney disease (CKD), chronic lung disease, and congestive heart failure.

A significant subgroup interaction for in-hospital mortality within the matched cohort according to age and comorbidity of chronic heart failure was observed in the subgroup analysis. Compared with older patients, those younger than 75 had a higher mortality with TAVR (Pinteraction = 0.03). Patients who are comorbid with congestive heart failure had a lower mortality with TAVR (Pinteraction < 0.001). No significant subgroup interaction was observed according to sex (Pinteraction = 0.63), CKD (Pinteraction = 0.86), or chronic lung disease (Pinteraction = 0.67) (Figure 4A).

Subgroup analysis for acute kidney injury within the matched cohort showed TAVR-associated risk reduction in all sex, age, CKD, congestive heart failure, and chronic lung disease subgroups; however, CKD and congestive heart failure comorbidity status showed significant subgroup interaction (Pinteraction < 0.001) (Figure 4B).

### Secondary Analysis Comparing TAVR Patients With or Without AF

From 2012 to 2016 NIS dataset, we generated a TAVR cohort including 107,375 hospitalizations: 45,540 (42.4%) had AF and 61,835 (57.6%) without AF. Using propensity score matching, we generated a cohort including 84,970 hospitalizations: 42,485 for TAVR with AF and 42,485 for TAVR without AF. Baseline characteristics before and after the matching of both groups are outlined in Table 3.

**Table 3:** Baseline characteristics for TAVR in patients with or without AF in Unmatched and Matched Cohorts.

	**Unmatched Cohort**		***P***	**Matched Cohort**	
	**TAVR in AF** ** (*n* = 45,540)**	**TAVR in No AF** ** (*n* = 61,835)**		**TAVR in AF** ** (*n* = 42,485)**	**TAVR in No AF** ** (*n* = 42,485)**
Age, yrs	81.9 ± 7.2	80.27± 8.3	<0.001	81.9 ± 7.2	81.9 ± 7.3
Female	46.1	47.8	0.05	46.1	46.5
Race			<0.001		
White	85.3	90.1		89.9	87.3
Black	5.2	2.7		2.7	4.7
Hispanic	4.8	3.1		3.1	4.3
Hypertension	75.5	76.8	0.02	75.4	75.6
Diabetes	25.2	25.4	0.65	25.3	25.5
Diabetes with chronic complications	8.3	9.6	0.001	8.2	8.2
Chronic lung disease	32.2	29.2	<0.001	31.6	18.2
Congestive heart failure	5.5	4.8	0.01	5.4	4.9
Chronic renal disease	35.9	32.6	<0.001	35.4	34.5
Anemia	21.7	21.4	0.65	21.6	21.3
Arthritis	4.2	4.6	0.13	4.2	4.4
Coagulopathy	19.8	17.9	<0.001	19.6	18.9
Hypothyroidism	19.4	18.4	0.06	19.3	19.1
Liver disease	2	2.7	<0.001	1.9	1.8
Obesity	13.2	15.2	<0.001	13.2	13
Weight loss	4.6	3.5	<0.001	4.5	4.2
Peripheral vascular disease	25.7	26.8	0.09	25.9	26.2
Pulmonary circulation disorder	1.9	1.2	<0.001	1.8	1.5
Teaching hospital	76.7	76.1	0.09	89.7	89.4
Rural location	0.9	0.7	0.22	0.9	0.6
Large hospital bed size	76.7	76.1	0.53	76.2	75.6
**Primary payer**					
Medicare/Medicaid	93	90.6	<0.001	93.2	93.2
Private insurance	7.3	5.2	<0.001	5.3	5.2
Non-elective admission	23.4	20.6	<0.001	23.4	22.5
0–25th percentile income	20.1	22	<0.001	20.9	21

*Values are mean ± SD or percentages*.

*AF, atrial fibrillation; SAVR, surgical aortic valve replacement; TAVR, transcatheter aortic valve replacement*.

Before propensity matching, AF was associated with a higher in-hospital mortality compared to patients without AF (3.1 vs. 2.6%; *p* = 0.04), higher incidence of acute kidney injury (18.1 vs. 13.1%; *p* < 0.001), new dialysis (1.5 vs. 0.9%; *p* < 0.001), permanent pacemaker implantation (11.9 vs. 6.4%; *p* < 0.001), blood transfusion (16.3 vs. 13.8%; *p* < 0.001), cardiac complication (9.9 vs. 6.9%; *p* < 0.001), acquired pneumonia (1.8 vs. 1.5%; *p* < 0.03), urinary tract infection (8.9 vs. 7.1%; *p* < 0.001), mechanical ventilation (2.1 vs. 1.6%; *p* < 0.001), gastrostomy (0.8 vs. 0.6%; *p* = 0.03), cardiac arrest (2.7 vs. 1.9%; *p* < 0.001), cardiac shock (3.3 vs. 2.2%; *p* < 0.001), more intermediate care discharge (28 vs. 19.8%; *p* < 0.001, longer median length of hospital stay [5 (IQR: 3–9) vs. 4 (IQR: 2–7)], and higher hospitalization cost (57,178 ± 29,547 vs. 53,267 ± 27,082; *p* < 0.001).

**Figure 5:**
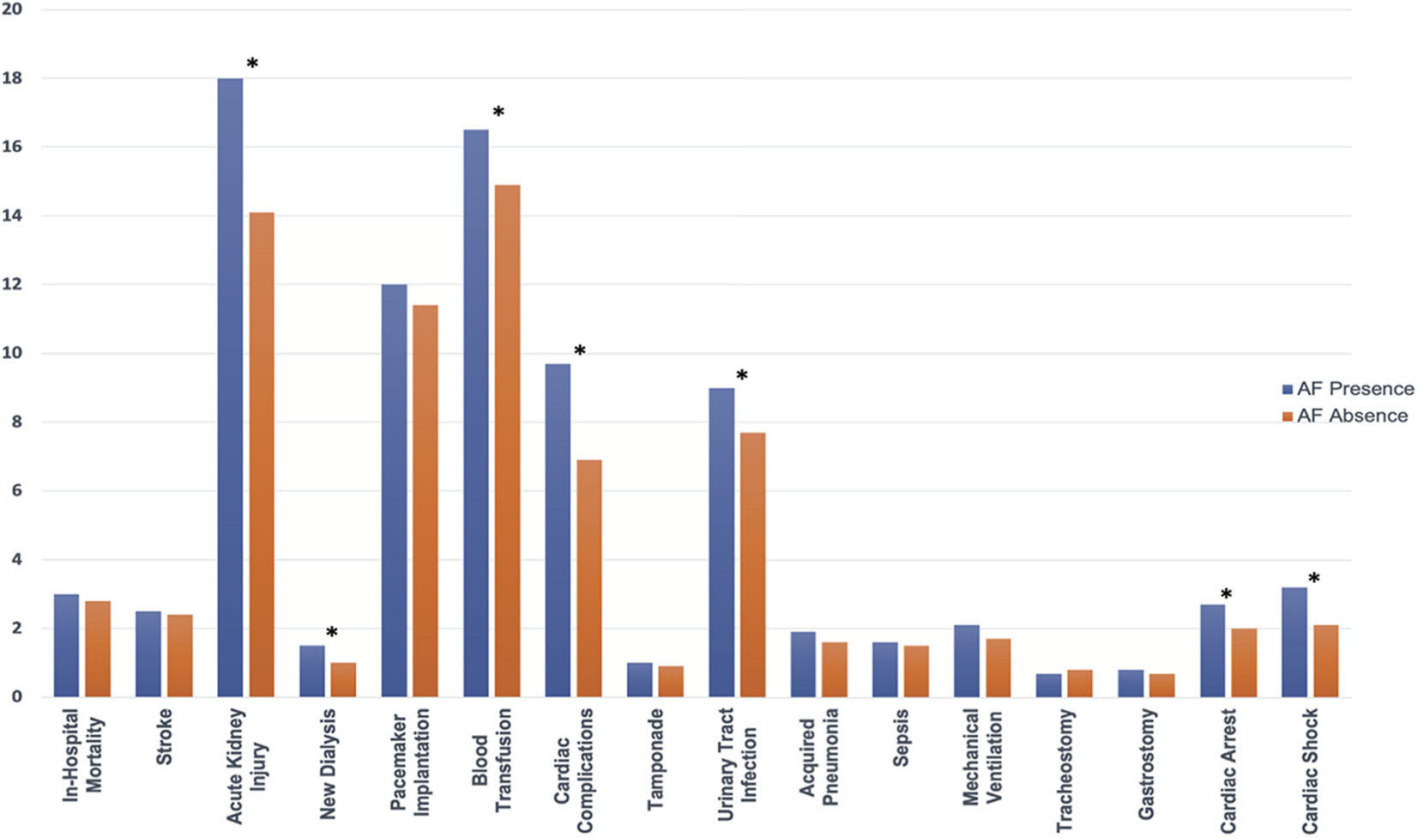
In-hospital outcomes comparing the presence and absence of AF in patients receiving TAVR among the matched cohort; abbreviations as in Figure 1. *indicate statistically significant determined by *P* < 0.05.

**Figure 6:**
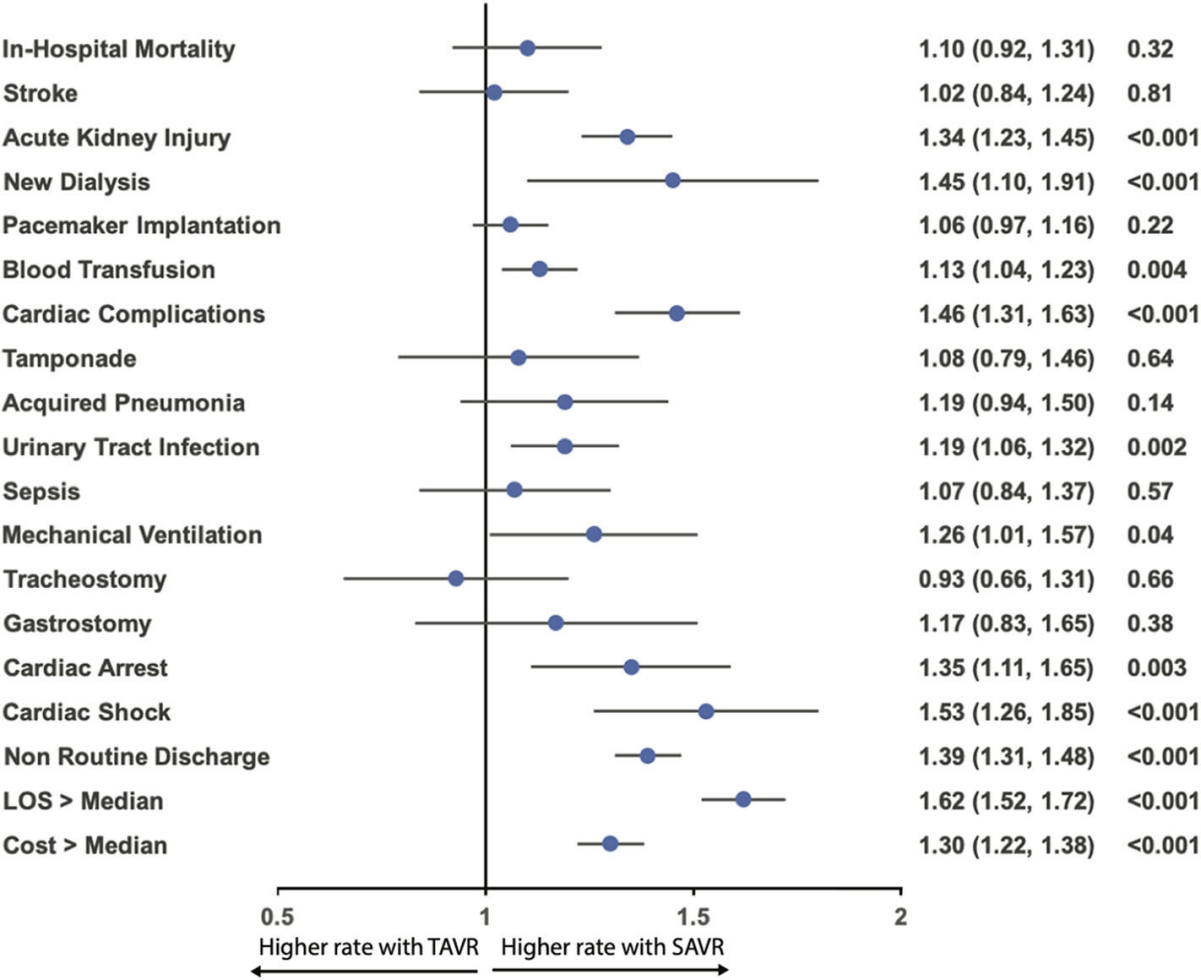
Odds ratio forest plot for in-hospital outcomes comparing the presence and absence of AF in patients receiving TAVR among the matched cohort. LOS, length of hospital stay; other abbreviations as in Figure 1.

After propensity matching, there was no difference between the patients with and without AF in the rate of in-hospital mortality (3.0 vs. 2.8%; OR:1.10; 95% CI: 0.92–1.31; *p* = 0.34), acute stroke (2.5 vs. 2.4%; OR:1.02; 95% CI: 0.84–1.24; *p* = 0.84), permanent pace maker implantation (12.0 vs. 11.4%; OR:1.06; 95% CI: 0.97–1.16; *p* = 0.23), tamponade (1.0 vs. 0.9%; OR:1.08; 95% CI: 0.79–1.46; *p* = 0.70), acquired pneumonia (1.9 vs. 1.6%; OR:1.19; 95% CI: 0.94–1.50; *p* = 0.16), sepsis (1.6 vs. 1.5%; OR:1.07; 95% CI: 0.84–1.37; *p* = 0.62), mechanical ventilation (2.1 vs. 1.7%; OR:1.26; 95% CI: 1.00–1.57; *p* = 0.05), tracheostomy (0.7 vs. 0.8%; OR:0.93; 95% CI: 0.66–1.31; *p* = 0.73), gastrostomy (0.8 vs. 0.7%; OR:1.17; 95% CI: 0.83–1.65; *p* = 0.43); however, AF was associated with a higher incidence of acute kidney injury (18.0 vs. 14.1%; OR:1.34; 95% CI: 1.23–1.45; *p* < 0.001), new dialysis (1.5 vs. 1.0%; OR:1.45; 95% CI: 0.1.10–1.91; *p* = 0.009), blood transfusion (16.5 vs. 14.9%; OR:1.13; 95% CI: 1.04–1.23; *p* = 0.003), cardiac complication (9.7 vs. 6.9%; OR:1.46; 95% CI: 1.31–1.63; *p* < 0.001), urinary tract infection (9.0 vs. 7.7%; OR:1.19; 95% CI: 1.06–1.32; *p* = 0.001), cardiac arrest (2.7 vs. 2.0%; OR:1.35; 95% CI: 1.11–1.65; *p* = 0.004), cardiac shock (3.2 vs. 2.1%; OR:1.53; 95% CI: 1.26–1.85; *p* < 0.001), more intermediate care discharge (27.6 vs. 21.4%; OR:1.39; 95% CI: 1.31–1.48; *p* < 0.001), longer length of hospital stay [5 (IQR: 3–9) vs. 4 (IQR: 2–7)], and higher hospitalization cost (57,214 ± 29,692 vs. 53,940 ± 28,237; *p* < 0.001) (Table 4; Figures 5, 6).

**Table 4:** Outcomes for patients with or without AF in the Unmatched and Matched Cohorts.

	**Unmatched Cohort**		***p***	**Matched Cohort**		***p***
	**TAVR in AF**	**TAVR in No AF**		**TAVR in AF**	**TAVR in No AF**	
In-Hospital death	3.1	2.6	0.04	3	2.8	0.34
Stroke	2.6	2.3	0.15	2.5	2.4	0.84
Acute kidney injury	18.1	13.1	<0.001	18	14.1	<0.001
New dialysis	1.5	0.9	<0.001	1.5	1	0.009
Pacemaker implantation	11.9	6.4	<0.001	12	11.4	0.23
Blood transfusion	16.3	13.8	<0.001	16.5	14.9	0.003
Cardiac complications	9.9	6.9	<0.001	9.7	6.9	<0.001
Tamponade	1.0	0.9	0.41	1	0.9	0.7
Acquired pneumonia	1.8	1.5	0.03	1.9	1.6	0.16
Urinary tract infection	8.9	7.1	<0.001	9	7.7	0.002
Sepsis	1.6	1.4	0.48	1.6	1.5	0.62
Mechanical ventilation	2.1	1.6	0.002	2.1	1.7	0.05
tracheostomy	0.7	0.7	1	0.7	0.8	0.73
Gastrostomy	0.8	0.6	0.03	0.8	0.7	0.43
Cardiac arrest	2.7	1.9	<0.001	2.7	2	0.004
Cardiac Shock	3.3	2.2	<0.001	3.2	2.1	<0.001
Discharge status			<0.001			<0.001
Routine	38.4	48.8		38.4	46.3	
Home Health Care	29.8	28.1		30.2	28.8	
Other Care Facility	28	19.8		27.6	21.4	
Length of stay, days	5 (3, 9)	4 (2, 7)	<0.001	5 (3, 9)	4 (2, 7)	<0.001
Mean cost, $	57,178 ± 29,547	53,267 ± 27,082	<0.001	57,214 ± 29,692	53,940 ± 28,237	<0.001

*Values are mean ± SD, median (interquartile range), or percentage*.

*AF, atrial fibrillation; SAVR, surgical aortic valve replacement; TAVR, transcatheter aortic valve replacement*.

## Discussion

The current study represents the largest propensity-matched cohort of patients with AF who underwent TAVR or SAVR. In this observational analysis including 186,744 hospitalizations, we compared in-hospital outcomes of TAVR vs. SAVR for patients with AF and in-hospital outcomes of TAVR for patients with AF vs. patients without AF. The primary study findings were as follows: (1) the number of AF patients receiving aortic valve replacement increases over the study period, and this trend is driven by the rising TAVR performance; (2) the in-hospital mortality for AF patients receiving TAVR declines during the study period, whereas that of those receiving SAVR remains unchanged; (3) the major predictor of TAVR for AF patients included age older than 75 years, comorbidity of congestive heart failure, chronic renal disease, pulmonary circulation disorder, teaching hospital admission, and Medicare/Medicaid insurance coverage; (4) compared to SAVR, although in-hospital mortality and acute stroke are similar, AF patients receiving TAVR had a lower rate of blood transfusion, acute kidney injury, new dialysis, cardiac complication, acquired pneumonia, sepsis, mechanical ventilation, tracheostomy, and gastrostomy as well as more routine discharge and shorter length of hospitalization stay; and (5) compared to TAVR without AF, cardiac transfusion, cardiac mortality, and acute stroke are similar, and the presence of AF was associated with increased incidence of blood transfusion, acute kidney injury, and dialysis. AF was also associated with more nursing facility discharge, a longer hospital stay, and higher hospital costs.

TAVR has revolutionized the clinical management of patients with AS. It was initially indicated for patient at excessively high risk of death or major complications to undergo SAVR. Following the PARTNER 3 and Evolut low-risk trials (7, 8), TAVR has become the standard of care for patients with severe AS across all risk groups. AF is the most common arrhythmia and increases with age. Using this large nationwide database, we confirmed that 45% of AS patients who received valve replacement were comorbid with AF. While the AF population that received SAVR remained unchanged over the study period, the AF population that received TAVR increased rapidly, which mirrored indication expansion and increased TAVR performance.

Our analysis shows that, compared with SAVR, patients who received TAVR are older and with more comorbidities. This explains the higher mortality in the TAVR group than in the SAVR group before matching. Importantly, the in-hospital mortality remains unchanged during the study period; however, in-hospital mortality for AF patients who received TAVR decreased dramatically during the study period. This observation can be seen as a combined effect of the valve, delivery device upgrade, accumulation of the TAVR operator experiences, and expansion of indication from inoperable to high- and intermediate-risk patients in the TAVR group. Currently, since more low-risk patients are receiving TAVR, we might expect an even lower in-hospital mortality with TAVR. After propensity matching, in line with prior studies (3, 5, 19), no difference in mortality and acute stroke between TAVR and SAVR was observed. Compared to SAVR, TAVR increased the risk of permanent pacemaker implantation, and had a higher hospitalization cost, but TAVR was found to be favorable over SAVR in multiple in-hospital outcomes, as shown in the current study, including blood transfusion, acute kidney injury, new dialysis, cardiac complications, acquired pneumonia, sepsis, mechanical ventilation, tracheostomy, and gastrostomy. Additionally, TAVR was associated with more routine discharge, less intermediate nursing facility discharge, and a much shorter length of hospital stay.

Subgroup analyses show that TAVR had higher mortality in patients younger than 75. Despite propensity score matching, younger AF patients who underwent TAVR were likely to have more comorbidity and are frailer, and surgeons were reluctant or refuse to offer SAVR, which may account for the increased mortality in the younger group. Consistent with prior studies (20, 21), the benefit of TAVR regarding the reduction in acute kidney injury occurs across all subgroups, although it is more evident in patients comorbid with CKD and congestive heart failure.

Secondary analysis showed, after propensity matching, there was no difference in in-hospital mortality and acute stroke between TAVR patients with AF and TAVR patient without AF. However, comorbidity with AF was associated with multiple in-hospital complications and increased health resource usage.

SOURCE XT analysis (10) showed that the presence of AF increases all-cause mortality at long-term follow-up, and new-onset AF was associated with increased risk of stroke. Our data showed that being comorbid with AF does not predispose them to excessive in-hospital mortality risk; thus, the excessive mortality and stroke risk are more likely derived from the long-term effect of AF. In the GALILEO trial, in patients without an established indication for anticoagulation after TAVR, oral anticoagulant rivaroxaban is detrimental rather than beneficial (22). A large proportion of AF AS patients are predisposed to ischemic stroke due to an aging population and comorbidity burdens, and data on whether and how the CHA_2_DS_2_-VASc score applies to TAVR AF patients are still lacking. The long-term optimal management of these AF patients remains challenging, especially because the optimal antithrombotic regimens following TAVR have yet to be determined for AF patients or certain subgroups of AF populations (23–26).

Our analysis is, to our knowledge, the largest cohort comparing TAVR and SAVR in the AF population (27, 28), considering the high prevalence of AF among AVR candidates. Our findings have significant public health implications. TAVR and SAVR had a similar impact on the incidence of in-hospital mortality and stroke. Although TAVR reduced multiple complications and reduced the length of hospital stay, it was associated with more permanent pacemaker implantation and increased hospital cost. AF should be seen as an important clinical factor to guide the clinician to decide on the best treatment options for individual patients. In patients receiving TAVR, AF patients suffer from more in-hospital complications and are associated with more resource usage; therefore, AF conditions warrant additional attention regarding the evaluation and optimal management of this population. Our study also showed that among patients who underwent TAVR, after propensity matching, in-hospital mortality and acute stroke are similar in patients with or without AF; therefore, the impact of AF on mortality is more likely related to the long-term effect of AF rather than playing an important periprocedural role. Thus, we should place more emphasis on the optimal long-term management of TAVR patients with AF.

## Limitations

The present analysis has certain limitations. Large inpatient cohorts, such as the NIS, are subject to coding and documentation errors. The administrative database lacks clinical details for each individual, and much important clinically relevant information, including echocardiography, laboratory data, and medications, could not be retrieved from the NIS database. Cardiac and valvular function are important variables for patients with AS, so we cannot incorporate these variables into the propensity score matching. In addition, aortic regurgitation and long-term data are unavailable. Additionally, retrospective observational analysis was liable to selection bias. However, the database has been validated internally and externally (29, 30). Moreover, we conducted robust analyses, including propensity matching and subgroup analysis, to reduce selection bias. Prior studies have suggested different clinical impacts regarding “pre-existing AF” and “new onset of AF” (10). Currently, studies have not distinguished new-onset AF from pre-existing AF; however, new-onset AF is often merely concealed at the time of hospitalization, and even genuine new-onset AF might have a common underlying pathophysiological mechanism with pre-existing AF. Further investigation with long-term follow-up is warranted for a better understanding of the impact of AF in aortic valve replacement patients.

## Conclusions

In this 5-years nationwide in-hospital dataset analysis, 45% of aortic valve replacement candidate were comorbid with AF. Among AF patients, compared to SAVR, TAVR was associated with multiple favorable in-hospital outcomes, resulting in shorter length of stay, and favorable discharge status. Therefore, TAVR was an appealing option for patients with AF. Nevertheless, the presence of AF markedly increased the risk for peri-procedure complications, associated with a lengthy hospital stay, unfavorable discharge status, and increased medical cost.

## Data Availability Statement

HCUP data is publicly available, but require proper training and purchase to gain access, data distribution can only be done by HCUP. users are not allowed to distribute the raw data, therefore the raw data could not be made publicly available by authors.

## Ethics Statement

Ethical review and approval was not required for the study on human participants in accordance with the local legislation and institutional requirements. Written informed consent for participation was not required for this study in accordance with the national legislation and the institutional requirements.

## Author Contributions

MZ, JW, and XC conceived the study. MZ, CL, and ZL performed data analysis. MZ drafted manuscript. YZ, QT, and QL reviewed and edited the manuscript. All authors contributed to the article and approved the submitted version.

## Conflict of Interest

The authors declare that the research was conducted in the absence of any commercial or financial relationships that could be construed as a potential conflict of interest.

## Abbreviations

AF: atrial fibrillation
AS: aortic stenosis
CKD: chronic kidney disease
CABG: coronary artery bypass grafting
ICD: international classification of diseases
NIS: national impatient sample
SAVR: surgical aortic valve replacement
TAVR: transcatheter aortic valve replacement.
