# Supplementary material for: Temporal Trends and Outcomes of Percutaneous and Surgical Aortic Valve Replacement in Patients With Atrial Fibrillation
Source: Front Cardiovasc Med. 2020 Dec 7;7:603834. doi: 10.3389/fcvm.2020.603834 (PMC7750195; doi:10.3389/fcvm.2020.603834)
Supplement: Supplementary file 1 [file Table_1.DOCX]

Supplementary Material

**Supplementary table 1**: List of the used ICD-9. ICD-10, and CCS codes. AF = atrial fibrillation; AVR = aortic valve replacement; CABG = coronary artery bypass grafting; SAVR = surgical aortic valve replacement; TAVR = transcatheter aortic valve replacement.

|  | | ICD 9 CODE | ICD 10 CODE |
| --- | --- | --- | --- |
| TAVR |  | 35.05, 35.06 | 02RF37H, 02RF38H, 02RF3JH, 02RF3KH, 02RF37Z, 02RF38Z, 02RF3JZ, 02RF3KZ |
| SAVR |  | 35.21, 35.22 | 02RF07Z, 02RF08Z, 02RF0KZ 02RF47Z, 02RF48Z, 02RF4JZ, 02RF4KZ, 02RF0JZ |
| AF |  | 427.31 | I48, I48.0, I48.1, I48.11, I48.19,  I48.2, I48.20, I48.21, I48.91 |
| Aortic stenosis |  | 395.0, 395.2, 396.0, 396.2, 424.1, 746.3 | [I06.0](https://www.icd10data.com/ICD10CM/Codes/I00-I99/I05-I09/I06-/I06.0), [I06.2,](https://www.icd10data.com/ICD10CM/Codes/I00-I99/I05-I09/I06-/I06.0)  [I08.0](https://www.icd10data.com/ICD10CM/Codes/I00-I99/I05-I09/I08-/I08.0), I35.0, I35.2, |
| CABG |  | 36.10, 36.11, 36.12, 36.13, 36.14,  36.15, 36.16, 39.61, 39.66 | CCS-44 |
| Mitral valve surgery |  | 35.23, 35.24 | 02UG07Z, 02NG0ZZ, 027G04Z, 02QG0ZZ, 02UG08Z, 02UG0JZ, 02UG0KZ, 025G0ZZ, 027G0DZ, 027G0ZZ, 02BG0ZX, 02BG0ZZ, 02RG07Z, 02RG08Z, 02RG0JZ, 02RG0KZ, 02VG0ZZ, 02CG0ZZ, 02WG07Z, 02WG08Z, 02WG0JZ, 02WG0KZ |
| Pulmonary valve surgery |  | 35.25, 35.26 | 02WH0KZ, 02WH07Z, 02WH0JZ, 027H04Z, 02RH08Z, 02TH0ZZ, 02RH0JZ, 02RH07Z, 02BH0ZZ, 02BH0ZX, 027H0ZZ, 027H0DZ, 025H0ZZ, 02UH0KZ, 02UH0JZ, 02UH08Z, 02UH07Z, 02QH0ZZ, 02NH0ZZ) |
| Tricuspid valve surgery |  | 35.27,35.28 | 02UJ0KZ, 027J04Z, 02NJ0ZZ, 02QJ0ZZ, 02UJ07Z, 02UJ08Z, 02UJ0JZ, 027J0DZ, 027J0ZZ, 02BJ0ZX, 02BJ0ZZ, 02RJ07Z, 02RJ0JZ, 02CJ0ZZ, 027J04Z, 02RJ08Z, 02RJ0KZ, 02WJ08Z, 02WJ0JZ, 02WJ07Z, 02WJ0KZ |
| Blood transfusion | | 99.01-99.09 | [30243N0](http://www.icd10data.com/ICD10PCS/Codes/3/0/2/4/30243N0), [30243N1](http://www.icd10data.com/ICD10PCS/Codes/3/0/2/4/30243N1), [30243P0](http://www.icd10data.com/ICD10PCS/Codes/3/0/2/4/30243P0), [30243P1](http://www.icd10data.com/ICD10PCS/Codes/3/0/2/4/30243P1), [30243H0](http://www.icd10data.com/ICD10PCS/Codes/3/0/2/4/30243H0), [30243H1](http://www.icd10data.com/ICD10PCS/Codes/3/0/2/4/30243H1), [30240N0](http://www.icd10data.com/ICD10PCS/Codes/3/0/2/4/30240N0), [30240N1](http://www.icd10data.com/ICD10PCS/Codes/3/0/2/4/30240N1), [30240P0](http://www.icd10data.com/ICD10PCS/Codes/3/0/2/4/30240P0), [30240P1](http://www.icd10data.com/ICD10PCS/Codes/3/0/2/4/30240P1), [30240H0](http://www.icd10data.com/ICD10PCS/Codes/3/0/2/4/30240H0), [30240H1](http://www.icd10data.com/ICD10PCS/Codes/3/0/2/4/30240H1), [30230H0](http://www.icd10data.com/ICD10PCS/Codes/3/0/2/3/30230H0), [30230H1](http://www.icd10data.com/ICD10PCS/Codes/3/0/2/3/30230H1), [30230N0](http://www.icd10data.com/ICD10PCS/Codes/3/0/2/3/30230N0), [30230N1](http://www.icd10data.com/ICD10PCS/Codes/3/0/2/3/30230N1), [30230P0](http://www.icd10data.com/ICD10PCS/Codes/3/0/2/3/30230P0), [30230P1](http://www.icd10data.com/ICD10PCS/Codes/3/0/2/3/30230P1), [30233N0](http://www.icd10data.com/ICD10PCS/Codes/3/0/2/3/30233N0), [30233N1](http://www.icd10data.com/ICD10PCS/Codes/3/0/2/3/30233N1), [30233P0](http://www.icd10data.com/ICD10PCS/Codes/3/0/2/3/30233P0),  [30233P1](http://www.icd10data.com/ICD10PCS/Codes/3/0/2/3/30233P1) |
| Acute stroke | | CCS-109 | CCS-CIR0220, CIR021,CIR024 |
| Acute kidney injury | | 584 | N170, N171, N172, N178, N179, N19, N990, R34, R944 |
| Hemodialysis | | 39.95, 585.6 | 5A1D00Z, 5A1D60Z |
| Cardiac complication | | 997.1, 42.30, 42.33, 370 | T8201XA, T8202XA, T8203XA,  T8209XA, T82222A, T82223A,  T82228A, T82867A, T82897A,  T82897A, I97710, I97790, I9788,  I9789, I9781, I9782 |
| Cardiac tamponade | | 423.3 | I314 |
| Cardiac arrest | | 427.5 | I46, I46.9, I46.2, I97.12, I97.71, I97.710, I97.711, I97.121 |
| Cardiogenic shock | | 785.51 | R570 |
| Permanent pacemaker | | 37.80, 37.83 | 02HK3JZ, 02H63JZ, 02HN0JZ, [02H60JZ](http://www.icd10data.com/ICD10PCS/Codes/0/2/H/6/02H60JZ), [02H60NZ](http://www.icd10data.com/ICD10PCS/Codes/0/2/H/6/02H60NZ), [02H63JZ](http://www.icd10data.com/ICD10PCS/Codes/0/2/H/6/02H63JZ), [02H63NZ](http://www.icd10data.com/ICD10PCS/Codes/0/2/H/6/02H63NZ), [02H64JZ](http://www.icd10data.com/ICD10PCS/Codes/0/2/H/6/02H64JZ), [02H64NZ](http://www.icd10data.com/ICD10PCS/Codes/0/2/H/6/02H64NZ), [02HK0JZ](http://www.icd10data.com/ICD10PCS/Codes/0/2/H/K/02HK0JZ), [02HK0NZ](http://www.icd10data.com/ICD10PCS/Codes/0/2/H/K/02HK0NZ), [02HK3JZ](http://www.icd10data.com/ICD10PCS/Codes/0/2/H/K/02HK3JZ), [02HK3NZ](http://www.icd10data.com/ICD10PCS/Codes/0/2/H/K/02HK3NZ), [02HK4JZ](http://www.icd10data.com/ICD10PCS/Codes/0/2/H/K/02HK4JZ), [02HK4NZ](http://www.icd10data.com/ICD10PCS/Codes/0/2/H/K/02HK4NZ), 02HN4JZ, 0JH604Z, 0JH634Z, 0JH605Z, 0JH607Z, 0JH635Z, [0JH606](http://www.icd10data.com/ICD10PCS/Codes/0/J/H/6/0JH606Z)Z, 0JH634Z, 0JH635Z, 0JH636Z, 0JH637Z |
| Acquired pneumonia | | 486, 481, 482.8, 482.3 | J13, J14, J15.1, J15.2, J15.4, J15.5, J15.6, J15.7, J15.8, J15.9, J16.0 |
| Sepsis | | 038, 995.91, 995.92, 996.64,  999.31, 999.32 | A410, A411, A412, A413, A414,  A415, A418, A419, A400, A401,  R6520, T8351XA, T80219A,  T80211A, A403, A408, A409,  B377, R651, T835, T857,  T827, T814 |
| Mechanical ventilation | | 96.72 | 5A1955Z |
| Tracheostomy | | 311, 31.21, 31.29 | 0B110F4, 0B110Z4, 0B113F4, 0B113Z4  0B114F4, 0B114Z4 |
| Gastrostomy | | 43.11, 43.19 | 0DH63UZ, 0DH64UZ, 0D16074,  0D160J4, 0D160K4, 0D160Z4,  0D163J4, 0D16474, 0D164J4,  0D164K4, 0D164Z4, 0D16874  0D168J4, 0D168K4, 0D168Z4  0DH60UZ |

## Supplementary Figures

Supplementary Figure 1: Trend in patient with AF and without AF receiving aortic valve replacement. (A) Trend in AF patients receiving AVR. (B) Trend in AF patients receiving SAVR. (C) Trend in AF patients receiving TAVR. AF = atrial fibrillation; AVR = aortic valve replacement; SAVR = surgical aortic valve replacement; TAVR = transcatheter aortic valve replacement.
